# Neural circuit and synaptic dysfunctions in ALS-FTD pathology

**DOI:** 10.3389/fncir.2023.1208876

**Published:** 2023-07-04

**Authors:** Santiago Mora, Ilary Allodi

**Affiliations:** ^1^Integrative Neuroscience Unit, Department of Neuroscience, Panum Institute, University of Copenhagen, Copenhagen, Denmark; ^2^Neural Circuits of Disease Laboratory, School of Psychology and Neuroscience, University of St Andrews, St Andrews, United Kingdom

**Keywords:** amyotrophic lateral sclerosis (ALS), frontotemporal dementia (FTD), synapses and neurons, motor control, cognitive functions

## Abstract

Action selection is a capital feature of cognition that guides behavior in processes that range from motor patterns to executive functions. Here, the ongoing actions need to be monitored and adjusted in response to sensory stimuli to increase the chances of reaching the goal. As higher hierarchical processes, these functions rely on complex neural circuits, and connective loops found within the brain and the spinal cord. Successful execution of motor behaviors depends, first, on proper selection of actions, and second, on implementation of motor commands. Thus, pathological conditions crucially affecting the integrity and preservation of these circuits and their connectivity will heavily impact goal-oriented motor behaviors. Amyotrophic Lateral Sclerosis (ALS) and Frontotemporal Dementia (FTD) are two neurodegenerative disorders known to share disease etiology and pathophysiology. New evidence in the field of ALS-FTD has shown degeneration of specific neural circuits and alterations in synaptic connectivity, contributing to neuronal degeneration, which leads to the impairment of motor commands and executive functions. This evidence is based on studies performed on animal models of disease, post-mortem tissue, and patient derived stem cells. In the present work, we review the existing evidence supporting pathological loss of connectivity and selective impairment of neural circuits in ALS and FTD, two diseases which share strong genetic causes and impairment in motor and executive functions.

## Introduction

Neurodegenerative diseases entail a major challenge as life expectancy increases in modern societies (Arthur et al., 2016). As one remarkable example, amyotrophic lateral sclerosis (ALS) is a fatal neurodegenerative disorder affecting motor neurons found in the spinal cord and brainstem, as well as corticospinal neurons. The disease is characterized by muscle wasting and progressive paralysis (Schweingruber and Hedlund, 2022). Frontotemporal dementia (FTD), instead, is a progressive condition characterized by the degeneration of neurons in the frontal and temporal lobes, leading to changes in behavior and personality, frontal executive deficits, and language dysfunctions. Depending on symptoms and etiology, FTD can be distinguished in its behavioral-variant FTD (bvFTD) or in two language variants, e.g., semantic variant primary progressive aphasia (svPPA), and non-fluent variant primary progressive aphasia (nfvPPA) (Butler and Chiong, 2019). ALS has an incidence of two per 100,000, with almost 120,000 new cases every year world-wide (Chio et al., 2013), while FTD is the second most common early onset dementia under 65 years of age (Harvey et al., 2003) with a conservative incidence of 1.6–4.1 cases per 100,000 per year (Butler and Chiong, 2019). An epidemiologic projection has shown that the number of ALS-FTD cases across the globe will increase from 222.801 in 2015 to 376.674 in 2040, which represents an increase of 69% (Arthur et al., 2016). It is now clear that ALS and FTD can overlap at genetic and pathological level, since genetic mutations in transactive response DNA-binding protein 43 (TARDBP), Fused-in-Sarcoma (FUS) and C9orf72 locus are associated with both disorders (Lomen-Hoerth et al., 2002; Renton et al., 2011; Al-Chalabi et al., 2012). ALS appear sporadically in 90% of cases (sALS), while in 10% of the cases the mutations are inherited (familial-fALS) (Al-Chalabi et al., 2012). Almost 50% of ALS cases develop FTD-like symptoms, while 20% of FTD cases develop ALS (Arthur et al., 2016). These discoveries have transformed the investigations on disease mechanisms; however, the origin and progression of ALS and FTD remain largely unclear and curative therapies do not exist. At a pathological level, dysfunctional cortical inhibition, leading to excitotoxicity, has been previously reported in both ALS and FTD (Vucic and Kiernan, 2006; Zhang et al., 2016). Our recent findings, obtained in the SOD1^*G*93*A*^ ALS mouse model, showed loss of spinal inhibitory inputs on motor neurons early in disease, preceding motor neuron death (Allodi et al., 2021). Imbalance between inhibition and excitation can lead to aberrant excitability, intracellular ion dysregulation and cell death, extensively reported in ALS-FTD (Roselli and Caroni, 2015; Jensen et al., 2020). Thus, maladaptive changes within neural circuits and alterations in connectivity might play a pivotal role in disease. An increasing number of studies performed on human post-mortem patients’ material, animal models of disease and patient-derived stem cells has reported alterations in synaptic functions in ALS-FTD (Allodi et al., 2021; Scekic-Zahirovic et al., 2021; Brown et al., 2022; Laszlo et al., 2022; Ma et al., 2022). Here, we summarize some of the latest findings while reviewing the neural circuits contributing to disease.

## Neural circuits affected in amyotrophic lateral sclerosis

Amyotrophic lateral sclerosis is characterized by progressive paralysis, hence progressive inability to perform movements. While degeneration of motor neurons is the immediate cause of this incurable disease, several cell non-autonomous mechanisms have been demonstrated to contribute to disease onset and progression in combination with the cell autonomous ones (for review Schweingruber and Hedlund, 2022). Here, we will focus on the contribution of intraspinal neurons as well as afferents and corticospinal descending inputs. The spinal cord is a hub for integration of inputs coming from supraspinal descending pathways, local interneuron circuits and sensory afferents. Spinal motor neurons are the ultimate output of the brain, since they directly connect to the muscles, however, within the spinal cord, they are activated by circuits of premotor neurons (Kiehn, 2006). Thus, execution of movements requires the synchronized or reciprocal activation of complex networks of inhibitory and excitatory spinal neurons (Kiehn, 2006). Spinal interneurons can be divided into ventral and dorsal populations depending on their location, and expression of lineage defined molecular markers and neurotransmitters (Briscoe et al., 2000; Lu et al., 2015). Dorsal populations relay somatosensory information coming from afferent pathways (Harding et al., 2020). Here, dorsal inhibitory and excitatory interneurons, as well as projection neurons send information from the spinal cord to the brain (Harding et al., 2020). Ventral interneurons are divided into V0 (V0_C_, V0_G_, V0_D_, and V0_V_), V1, V2 (V2a and V2b), and V3 cardinal populations. These interneurons regulate different aspects of locomotion, e.g., flexor-extensor coordination (Zhang et al., 2014), muscle force (Zagoraiou et al., 2009), left-right alternation (Crone et al., 2009; Dougherty et al., 2013; Talpalar et al., 2013; Bellardita and Kiehn, 2015) and speed of gait (Gosgnach et al., 2006). Molecular marker-specific expression allows for targeted silencing or depletion of interneuron populations leading to motor alterations and allowing identification of interneuron specific functions (Gosgnach et al., 2006; Zagoraiou et al., 2009; Talpalar et al., 2013; Zhang et al., 2014; Bellardita and Kiehn, 2015). Silencing of inhibitory V1 and V2b neurons disrupts the flexor-extensor muscle reciprocal recruitment (Zhang et al., 2014), while V1 silencing alone reduces the speed of locomotion and decreases stride length (Gosgnach et al., 2006; Allodi et al., 2021). Depletion of V0 commissural neurons causes the loss of left-right alternation of the limbs (Talpalar et al., 2013; Bellardita and Kiehn, 2015), as well as silencing of V2a interneurons perturbs the left-right alternation and the rhythm of locomotion (Crone et al., 2009; Dougherty et al., 2013).

Spinal interneuron loss in human post-mortem ALS tissue was first described in 1895 by Marie Pierre in its “Lectures on diseases of the spinal cord” (London, The New Sydenham). Quantitative studies reported reduction of interneurons in the ventral and intermediate areas of the spinal cord of ALS subjects (Oyanagi et al., 1989; Terao et al., 1994). Moreover, anatomical analysis in sporadic ALS patients showed similar loss of interneurons (56%) and motor neurons (64.4%) within the ventral horn of the spinal cord (Stephens et al., 2006). Not all motor neurons within the spinal cord are equally vulnerable to ALS degeneration. α motor neurons, innervating muscle fibers, degenerate in disease while γ motor neurons, innervating muscle spindles, are spared (Lalancette-Hebert et al., 2016). Among α motor neurons, the ones innervating slow-twitch (S) muscle fibers are more resistant compared to those innervating the fast twitch fatigable (FF) ones (Pun et al., 2006). Spinal motor neurons are known to become hyperexcitable already at presymptomatic stages (Jensen et al., 2020). Our recent findings demonstrated that, under physiological conditions, FF motor neurons receive stronger inputs from the V1 inhibitory population, found in the ventral and intermediate areas of the spinal cord and positive for the transcription factor Engrailed-1 (En1) (Allodi et al., 2021). We also showed that these interneurons are affected in presymptomatic SOD1^*G*93*A*^ mice, leading to preferential loss of inhibitory inputs on FF motor neurons. Loss of V1 synaptic inputs paralleled the onset of locomotor phenotype which is characterized by loss of speed, reduction of stride length and step frequency, as well as hyperflexion of the limbs (Allodi et al., 2021). These symptoms are also reported during early stages of disease in ALS patients (Radovanovic et al., 2014). This loss of synaptic inputs onto FF motor neurons can contribute their imbalanced excitability (Roselli and Caroni, 2015; Jensen et al., 2020). Reduction of V1 inhibitory inputs was reported also in a SOD1 zebrafish model (McGown et al., 2013). V1 interneurons are 80% glycinergic and among them are Renshaw cells and Ia inhibitory interneurons (Alvarez et al., 2005); a reduction of their synaptic inputs onto motor neurons was previously observed in presymptomatic (Chang and Martin, 2009, 2011) as well as in symptomatic mice (Salamatina et al., 2020). Moreover, inhibitory interneuron loss in the intermediate area and dorsal horn of the spinal cord was also reported in a BAC C9ofr72 mouse model (Liu et al., 2016). Interestingly, a recent electrophysiological study reported that, in the early postnatal SOD1*^G93A^* mice, glycinergic spinal interneurons were less excitable, showed smaller somas and larger neurites compared to control mice (Cavarsan et al., 2023). Moreover, the glycinergic interneurons most affected in disease, were the most ventrally located (putative Renshaw cells) and the ones within lamina IX (putative Ia) (Cavarsan et al., 2023). Thus, inhibitory interneuron presymptomatic changes can contribute to ALS pathology.

Intraspinal excitatory interneurons seem to degenerate at later stages during disease progression. Reduced synaptic inputs on lumbar spinal motor neurons was reported excitatory in the symptomatic FUS-R521C mouse model (Qiu et al., 2014). In the SOD1^G93A^ mouse, degeneration of V2a excitatory neurons, positive for the transcription factor Chx10 and providing vGlut2 positive inputs, was observed at later symptomatic stages (Romer et al., 2017; Salamatina et al., 2020). C-boutons are specialized synapses known to modulate motor neuron activity (Zagoraiou et al., 2009) and originate from the V0_c_ cholinergic interneurons positive for Dbx1 and Ptx2 (Zagoraiou et al., 2009). C-boutons were described to undergo structural changes in ALS patients (Nagao et al., 1998) and mouse models of disease (Herron and Miles, 2012; Landoni et al., 2019; Bak et al., 2023). Also, selective loss of tripartite synapses was observed in ALS post-mortem spinal cords as well as the SOD1^G93A^ mice spinal cords (Broadhead et al., 2022). Here, astrocytes regulate synaptic transmission by forming tripartite specialized connections, which were found preferentially affected within excitatory synapses (Broadhead et al., 2022). Thus, intraspinal circuits and their connection to motor neurons are affected in ALS throughout disease progression. Evidence from ALS mice suggests that intraspinal inhibitory interneurons might play a role early in disease, while intraspinal excitatory interneurons seem to be affected at later stages.

Depression of afferent inputs, known to provide excitation to motor neurons, has been previously reported in ALS patients (Sangari et al., 2016). Alterations in afferent inputs can contribute to changes in motor neuron excitability. Ia proprioceptive afferents provide a large proportion of vGlut1 positive synapses on α motor neurons (Alvarez et al., 2011) and are fund in higher density on FF motor neurons (Basaldella et al., 2015). Significant loss of vGlut1 synapses was found in symptomatic SOD1^G93A^ and TDP43^A315T^ mice (postnatal day 90) but not at presymptomatic stage (Vaughan et al., 2015). However, electrophysiological studies showed presymptomatic impairment of the excitatory connectivity between Ia afferents and motor neurons, as well as disruption of the vGlut1 postsynaptic density in SOD1 mice (Baczyk et al., 2020). Moreover, inhibition of Ia afferents and spared γ motor neurons was found protective in SOD1^G93A^ mice (Lalancette-Hebert et al., 2016). These data suggest dysfunctions of excitatory afferent inputs early in disease.

Cortical hyperexcitability is a known hallmark of the disease and has been reported in ALS patients (Vucic and Kiernan, 2006). Pyramidal neurons within layer V (also referred to as upper motor neurons) which form the corticospinal tract and project to the spinal cord are affected in disease (Vucic and Kiernan, 2006). Studies performed in ALS mouse models, suggest that this event contributes to the early stages of ALS degeneration, which triggers changes within the spinal cord (Marques et al., 2021; Reale et al., 2023). Interestingly, corticospinal neurons and spinal motor neurons seem to degenerate in a somatotopically related manner (Marques et al., 2021). Motor cortex specific mislocalization of TDP-43 led to cortical hyperexcitability and spinal motor neuron loss selectively in the lumbar segments 1–3 (Reale et al., 2023), the spinal cord segments where the central pattern generators supposedly located (Kiehn, 2006).

In ALS, main efforts have been directed to improve motor neuron survival and muscle reinnervation. However, it is now clear that loss of synapses happens not only in the peripheral nervous system, but also at different levels within the central nervous system. Our recent work shows that rescue of synaptic connectivity between V1 interneurons and motor neurons leads to increased number of spared motor neurons and amelioration of motor phenotype (Mora et al., 2022). Moreover, other studies point towards synaptic connectivity being a therapeutic target for treatment (Baczyk et al., 2020; Catanese et al., 2021). Hence, further investigations on changes in synaptic connectivity in intraspinal, afferent and corticospinal circuits could play an important role in deepening disease understanding and finding new targets for treatment.

## Neural circuits affected in frontotemporal dementia

Frontotemporal dementia is characterized by alterations in behavioral domains, including executive functions and social interactions. These symptoms generally appear subtle early in disease, leading to underdiagnosis of the condition. As mentioned above, FTD is found in comorbidity with ALS, due to mutations in the C9orf72, FUS, and TDP-43 genes (Lomen-Hoerth et al., 2002; Al-Chalabi et al., 2012). However, FTD can occur independently from ALS and liked to mutations in the progranulin (GRN) and microtubule-associated tau protein (MAPT) genes among others (Couratier et al., 2017; Butler and Chiong, 2019). In FTD, complex behavioral symptoms are paralleled by complex pathophysiological changes in cortical and subcortical circuits of the brain. Positron emission tomography enabling synaptic density quantification in bvFTD studies, showed severe synaptic loss of multiple brain regions including, the medial and dorsolateral frontal regions of the brain, the inferior frontal gyri, the anterior and posterior cingulate gyrus, the insular cortex, and the medial temporal lobe (Malpetti et al., 2023), here, synaptic loss was more severe than overall brain atrophy (Malpetti et al., 2023). Moreover, lower levels of synaptic proteins such as synaptotagmins and synaptophysin were observed years before disease onset in FTD patients (Goetzl et al., 2016).

**FIGURE 1:**
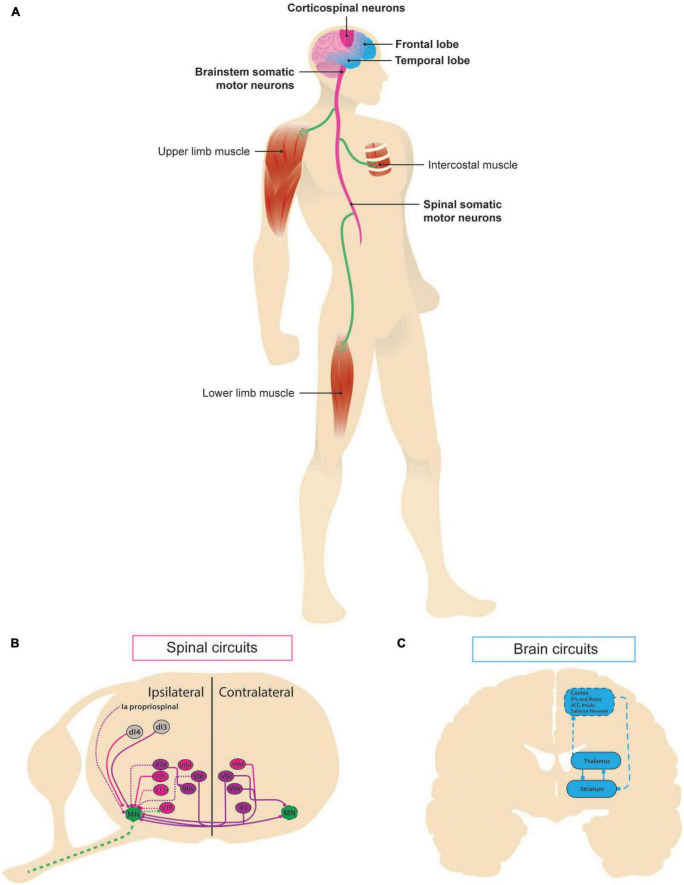
Neural circuits involved in ALS-FTD. Schematic representation of the circuits associated with amyotrophic lateral sclerosis (ALS) and frontotemporal dementia (FTD) as manifestations of the same continuum. **(A)** In ALS, pathological changes can be first seen in the brainstem somatic motor neurons, whose death results in upper-body phenotype, known as “bulbar onset,” or in spinal somatic motor neurons, whose death leads to lower limbs symptomatology in the so-called “lumbar onset,” along with death of the corticospinal neurons of the motor cortex. Affected areas are shown in dark pink. FTD affects the frontal and temporal lobes of the brain. Areas contributing to the disease are depicted in light blue. **(B)** Schematic of intraspinal lumbar circuits known to play a role in motor control. Spatial localization of ventral cardinal populations (V0_C_, V0_D_, V0_V_, V1, V2a, V2b, and V3) and some dorsal populations. Inhibitory (pink) and excitatory (purple) populations and inputs are depicted, dashed lines indicate known structure degeneration. **(C)** At a systems level, frontotemporal dementia has been linked to altered corticostriatal circuits [adapted from Dalley et al. (2011) and Radulescu et al. (2017)]: cortex sends excitatory glutamatergic projections to the medium spiny neurons of striatum expressing dopamine receptor 1 and 2, which inhibit each other and the internal and external, respectively, regions of globus pallidus via GABAergic activity. Its external part sends inhibitory projections to subthalamic nucleus (not shown), which in turn sends excitatory input back the internal part, which inhibits thalamus. Finally, thalamus sends excitatory projections back to striatum and cortex.

The C9orf72 pathology is characterized by hexanucleotide repeat expansion in the first intron of the C9orf72 gene, a common ALS-FTD mutation that results in the formation of nuclear RNA foci, dipeptide repeat protein inclusions and TDP43 aggregates and is the most frequent reported genetic cause of ALS-FTD (Renton et al., 2011). Studies performed on synaptoneurosomes obtained from ALS post-mortem human cortex carrying C9orf72 hexanucleotide repeat expansion revealed extensive synaptic dysfunction for both excitatory and inhibitory synapses (Laszlo et al., 2022). Mice carrying C9orf72 hexanucleotide repeat expansion show abnormal social interaction, hyperactivity, anxious phenotype and, in some cases, motor deficits (Chew et al., 2015; Jiang et al., 2016; Liu et al., 2016) and memory impairment (Jiang et al., 2016). Anatomical studies performed on these mice have reported lower neuron count within the cortex (especially the motor areas) and the cerebellum, paralleled with reactive gliosis (Chew et al., 2015; Jiang et al., 2016; Liu et al., 2016). Reduction of hippocampal neurons was also shown in two BAC C9orf72 transgenic mouse lines (Jiang et al., 2016; Liu et al., 2016), while only one of them in spinal motor neuron and interneuron loss was observed (Liu et al., 2016). However, while neuronal loss has been more thoroughly investigated, less is known about synaptic connectivity in the C9orf72 mouse models.

**TABLE 1:** Altered biomarkers underlying clinical symptomatology.

	Structure	Clinical output	Animal model				
			*C9ORF72*	*TDP-43*	*FUS*	*Tau*	*GRN*
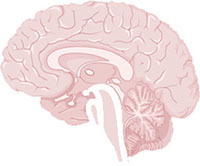	Frontal cortex	Apathy		↓ Synaptic activity and formation; gene expression	●↓ Synaptic connectivity ●↓ Dendritic spine number ●↓ EPSCs amplitude ●↑ neuronal activity		↓ Late dendritic arborization
		Dietary shifts						
	Striatum	Compulsive behavior				●↓ NMDAR synaptic density ●↓ firing	↑ Excitability of spiny neurons
	Motor cortex	Stereotypies	Reactive gliosis	●Hyperactive Sst neurons ●↓ GABA transmission ●↑ Excitability, excitotoxicity	●↑ Excitatory synapses markers ●↓ Inhibitory synapses markers ●↓ Dendritic length		
	Hippocampus	Memory deficits	↓ Neuron count	●↑ Microglial activity ●↓ Ventral mature and precursor granular cells	●↓ Synaptic connectivity ●↓ Dendritic spine number	●↓ Ventral mature and precursor granular cells ●↑ GABAergic innervation	●↑ Microglial activity ●↓ Synaptic connectivity ●↓ LTP ●↓ Dendritic length and spine density and number
	**IN type**	**Function**	**Animal model**				
			***C9ORF72***	***SOD1***	***TDP-43***	***FUS***	
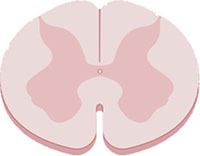	V0c	Muscle tone		Early enlargement, then loss of synapses			
	V2a	Rhythm of locomotion		●↓ Chx10+ neuron count ●↓ Synapses			
	V1	● Speed of locomotion ● Stride length		●↓ En1+ neuron count ●↓ Synapses			
	*Not specified		↓ Interneuron count	↓ Excitatory synapses (vGlut1)	●↑ excitatory synapses (vGlut2) ●↑ inhibitory synapses (GAD65/67)	↓ excitatory synapses	

It summarizes the link between the aforementioned pathophysiological alterations in ALS-FTD by brain area and relevant symptomatology associated with alterations of such regions. Although more or less defined links between brain pathology and clinical outcomes are stated for the sake of simplicity, future studies should aim to investigate the phenomenon from a circuit neuroscience perspective given the nosological complexity of it and the roles of these structures in more than one behavior under normal conditions, especially when taking into account fronto-striatal circuits (Dalley et al., 2011).

TDP-43 and FUS play an important role in regulating strength of synaptic transmission (for review Ling, 2018). Gene expression analysis in FTD post-mortem human tissue with TDP-43 pathology reported downregulation of genes involved in synaptic activity and synapse formation (Mishra et al., 2007). Similar results were obtained by cross-linking and immunoprecipitation coupled with high-throughput sequencing performed in the brain of a mouse model upon reduction in TDP-43 (Polymenidou et al., 2011). Several of the generated TDP-43 transgenic mice recapitulate ALS-FTD pathology and symptoms (among others Wegorzewska et al., 2009; Wils et al., 2010; Swarup et al., 2011). Here, layer V pyramidal neurons in the frontal, motor and somatosensory corteces, spinal motor neurons (Wegorzewska et al., 2009; Wils et al., 2010; Igaz et al., 2011) as well as hippocampal neurons (Igaz et al., 2011) were found preferentially affected. In a TDP-43 transgenic mouse, hyperactive somatostatin positive interneurons were shown to indirectly disinhibit layer V pyramidal neurons leading to excitotoxicity (Zhang et al., 2016). Severe cognitive impairment within the learning and memory domains was reported in TPD-43 transgenic mice (Swarup et al., 2011), also when TDP-43 overexpression was restricted to the forebrain (Tsai et al., 2010). Here, progressive motor dysfunctions were also observed (Tsai et al., 2010). FUS is an important protein involved in the processing of coding and non-coding RNA, and in the maintenance of genomic integrity. Mutations in the FUS gene induce pathological cytoplasmic mislocalization of the protein, especially within the stress granules, which can be observed in human post-mortem tissue and FUS mouse models, in absence of TDP-43 pathology (Nolan et al., 2016). FUS pathology has been reported to affect neurons within frontal and temporal neocortex, hippocampus, and striatum (Mackenzie et al., 2011). Several FUS transgenic mice were generated, however, not all of them showed neuronal degeneration even in presence of cognitive and motor dysfunctions (for review Nolan et al., 2016). Changes in synaptic connectivity and dendritic spine morphology were described in the sensorimotor cortex (Qiu et al., 2014). In the Fus^ΔNLS/+^ mouse, increase of FUS at synaptic level was observed early in disease and led to changes in density and size of GABAergic synapses (Shiihashi et al., 2017; Sahadevan et al., 2021). The Fus^ΔNLS/+^ transgenic mice showed increased activity in the layers II/III of the frontal cortex and overall reduced brain volume, which were paralleled by increased social interaction and hyperactivity, along with worse learning and memory (Shiihashi et al., 2017; Scekic-Zahirovic et al., 2021).

Other known FTD-linked mutations are found in the progranulin (GRN) and microtubule-associated tau protein (MAPT) genes. GRN deficiency leads to progranulin haploinsufficiency, and has been linked to several behavioral impairments and central nervous system alterations (Roberson, 2012). Here, top-down cognitive control is impaired (Dalley et al., 2011), and cortico-striatal-thalamic alterations are commonly reported. GRN transgenic mouse models show decreased synaptic connectivity and impaired plasticity (Petkau et al., 2012; Lui et al., 2016). Changes in dendritic arborization and electrophysiological alterations in prefrontal regions, amygdala and thalamus were also reported (Arrant et al., 2016; Lui et al., 2016; Nagy et al., 2019). Phenotypically, dysfunctional social interaction and motor coordination (Petkau et al., 2012; Arrant et al., 2016) were shown, as well as impairment of executive functions leading to excessive grooming, deficient exploration of novel objects, and increased reward seeking (Petkau et al., 2012; Lui et al., 2016; Nagy et al., 2019). Tau pathology also leads to protein accumulation and is linked to behavioral and neurophysiological changes. Transgenic mouse models show degeneration in the hippocampus and increased GABAergic inhibition (Llorens-Martin et al., 2011), as well as NMDA synaptic deficits in the ventral striatum and insula (Warmus et al., 2014). Alterations in executive function and emotional processing were also reported, such as compulsive grooming and increased immobility (Llorens-Martin et al., 2011; Warmus et al., 2014). Altogether, these findings support the idea that alterations in synaptic connectivity within specific neural circuits play a pivotal role in FTD pathophysiology.

## Discussion

This minireview focuses on the existing evidence that several neural circuits are affected in ALS-FTD pathology. Here, results obtained from ALS-FTD human post-mortem tissue and mouse models of disease suggest that changes in synaptic connectivity and subsequent imbalance between inhibitory and excitatory networks might play a role, together with other pathological events, at different stages of disease. Imaging studies suggest that FTD affects the *salience network*, which includes multimodal networks identifying salient information (Roberson, 2012). The salience network is found in the anterior cingulate and ventral anterior insular cortices, and it is phylogenically conserved between mice and men (Roberson, 2012). Thus, while in humans, ALS and FTD are characterized by the degeneration of Betz cells and von Economo neurons within the prefrontal and frontal cortices, which are not present in mice, network activity and degeneration can still be mimicked in mouse models. Cell types are not the only non-conserved feature among species, the corticospinal tract is also known to differ anatomically and functionally between humans and mice. Moreover, intraspinal neural networks controlling different aspects of motor control and forming the central pattern generators are under investigated in the human spinal cord, although new comparative studies points toward maintenance of spinal neuron molecular markers (Yadav et al., 2023) and functions (Kathe et al., 2022). Overall, further investigations are needed to deepen our understanding of neural networks alteration in ALS-FTD and their contribution to neuronal vulnerability in disease.

## Author contributions

IA: conceptualization and funding acquisition. Both authors contributed to writing - original draft, figure, table and the article and approved the submitted version.
